# Mental Health Benefits of Maitri Sambodh Dhyaan (MSD) Meditation for Dementia Caregivers

**DOI:** 10.1093/geroni/igaf122.1047

**Published:** 2025-12-31

**Authors:** Nirmala Lekhak, Tirth Bhatta, Cecilia Fernandes, Karenna Tran, Santosh Gupta

**Affiliations:** University of Nevada, Las Vegas, Las Vegas, Nevada, United States; University of Nevada, Las Vegas, Las Vegas, Nevada, United States; PrismaHealth Children’s Hospital, Columbia, South Carolina, United States; University of Nevada, Las Vegas, Las Vegas, Nevada, United States; P.D. Hinduja Hospital, Mumbai, Maharashtra, India

## Abstract

Prior research has consistently established the role of meditation as a coping resource to mitigate the adverse impacts of stressful life situations on mental health. However, existing research on positive effects and mechanisms that transmit these positive effects of meditation for current and former dementia caregivers is limited. This qualitative study is focused on how current and former dementia caregivers perceive effects of Maitri Sambodh Dhyaan (MSD) meditation, a unique practice that integrates elements of previously researched meditation techniques into one. We used longitudinal qualitative descriptive content analysis of open-ended questions asked of 44 current and former dementia caregivers. The MSD meditation intervention was administered for 21 days and after 21 days the participants were asked to practice on their own with the video recording that was provided to them. We followed up in 30- and 60-days. Positive emotions that dementia caregivers experienced after their practice of meditation included calmness, relaxation, refreshing, content, happy, peaceful, focused, connectedness, appreciative for the meditative time and intervention. The participants also reported improvement in their self-care, mental health, sleep quality, and reaction to their care-recipient. The findings highlight the importance of integrating meditation practices into caregiver support programs to strengthen emotional resilience, enhance mental health, and improve coping mechanisms.

